# Construction of Hierarchical Fe-MFI Nanosheets with Enhanced Fenton-like Degradation Performance

**DOI:** 10.3390/molecules30194030

**Published:** 2025-10-09

**Authors:** Haibo Jiang, Lin Xu, Qingrun Meng, Xu Feng, Junxuan Wang, Yankai Li, Junjie Li

**Affiliations:** 1 Key Laboratory of Energy Chemical and Nano-Catalysis of Liaoning Province, School of Chemical and Environmental Engineering, Liaoning University of Technology, Jinzhou 121001, China; jhb06925@gmail.com (H.J.); 15102457917@163.com (L.X.); hgfengxu@163.com (X.F.); 18756217591@163.com (J.W.); a13130329878@163.com (Y.L.); 2 Dalian Institute of Chemical Physics, Chinese Academy of Sciences (CAS), Dalian 116023, China

**Keywords:** Fenton-like, hierarchical zeolite, Fe-MFI nanosheets, alkali treatment, advanced oxidation

## Abstract

Introducing hierarchical structure into zeolites or synthesizing two-dimensional (2D) zeolite nanosheets have drawn much attention in catalysis and separation process due to the improvement in zeolites’ diffusion properties. In this study, Fe incorporated on the MFI zeolite framework (Fe-MFI) with the nanosheet morphology and unique hierarchical pore structure was successfully synthesized and applied for the adsorption and degradation of Rhodamine B (RhB) in a Fenton-like reaction in the presence of H_2_O_2_. The synthesis involved a seed-directed hydrothermal method in the presence of NH_4_F and a subsequent NaOH treatment made the synthesized hierarchical Fe-MFI nanosheets (Fe-20-10) characterized by abundant highly dispersed framework Fe^3+^ species. As a result of these features, the Fe-20-10 showed excellent ability of adsorption and degradation efficiency of RhB, and enhanced durability due to negligible leaching of framework Fe^3+^ species. Moreover, the hydroxyl radicals were determined as the main the reactive oxygen species of RhB degradation, and a possible adsorption–degradation pathway was proposed. This work offers guidance for developing high-performance Fenton-like degradation catalysts.

## 1. Introduction

Water pollution caused by the discharge of various organic compounds (organic dyes, pharmaceuticals, pesticides, steroid estrogens, and personal care products) has become more serious and attracted public attention [1,2]. Rhodamine B (RhB), as a typical organic dye, is resistant to natural degradation due to its stable chemical structure. Undegraded RhB accumulated in water bodies poses significant health risks, being potentially carcinogenic and toxic, which can lead to organ damage upon exposure [3]. Different technologies have been applied to wastewater treatment, which can be divided into physical, chemical, biological, and combined processes [4,5]. Among them, chemical technology, especially for the advanced oxidation processes (AOPs) have emerged as a research focus due to their strong oxidation capacity, rapid degradation rates, and thorough pollutant mineralization [6]. As a key component of AOPs, Fenton/Fenton-like catalysis exhibits significant potential in wastewater treatment and environmental remediation, highly oxidative hydroxyl radicals (·OH) generate during this process, which effectively larger degrade refractory organic pollutants [7]. The conventional homogeneous Fenton process employs Fe(II) as the catalyst and H_2_O_2_ as the oxidant under acidic conditions; highly reactive hydroxyl radicals (·OH) were formed and Fe(II) was oxidated to Fe(III). Then, ·OH can react with organic compounds in a rapid free-radical chain reaction, which non-selectively mineralizes the organic compounds into harmless products such as CO_2_ and H_2_O [8]. However, the conventional homogeneous Fenton reaction usually faces challenges such as narrow reaction pH range (typically pH < 3), difficulty in separating and recycling, and poor stability of catalyst [9]. To address these drawbacks, various heterogeneous Fenton-like catalytic technologies have been developed. Fenton-like catalysis is defined as a process where Fe^3+^ or other transition metal ions replace Fe^2+^, and Fe^3+^ is mainly utilized in an insoluble form [10,11]. This technology mainly focuses on iron-based catalysts due to its high efficiency and recyclability [12]. Unfortunately, the lower hydroxyl radical generation rate, compared with the classical Fenton process and the leaching of Fe species, become an obstacle to its application; therefore, developing more efficient and stable Fe-based heterogeneous catalysts is of great significance.

Currently, there are approximately three types of Fe-based catalysts that have been widely reported: (i) single Fe-based oxides or natural Fe-containing materials, such as α-Fe_2_O_3_ (hematite), α-and γ-FeOOH (goethite and lepidocrocite), and Fe_3_O_4_ (magnetite) [13]; (ii) Fe-doped mixed oxides or carbon materials, such as Fe-CeO_2_, CuFeO_2,_ and Fe-doped carbon xerogel [14,15]; (iii) Fe-immobilized materials [16], which can be divided into supported (Fe supported on carbon, silica, Al_2_O_3_, and zeolites) [17,18], encapsulated (Fe-immobilized in resin, Nafion membrane, polyethylene film, carbon xerogel and so on) [19], and framework-stabilized (Fe was incorporated into the framework of MOFs or zeolite) Fe-based materials [20,21]. Comparatively, type (i) catalysts usually presented a better catalytic performance but a high leaching or dissolution of Fe species in the acidic reaction solution, while the resistance to leaching was significantly improved for the latter two, which also presented the comparable degradation efficiency of the type (i) catalysts. In particular, type (iii) catalysts have become more important in recent years. The crystalline porous zeolite materials with the advantages of high thermal and hydrothermal stability, finely tuned acid–base sites and hydrophilicity–hydrophobicity, diverse and stable framework structure, and excellent ion exchange-ability appear to be the most promising supports or host materials to stabilize metal particles [22]. Impregnation, ion exchange, and hydrothermal methods are the well-known methods for preparing zeolite-based Fenton-like catalysts [23]. However, the impregnation or ion exchange methods may reduce the external and internal specific surface area of zeolite, meanwhile the metal species are easily agglomerated and leached out, resulting in a great decrease in degradation performance [24]. Incorporation of active metal species (Fe) into the zeolite framework by the hydrothermal method can solve the above problems [25]. In addition, the intrinsic microporous structure of the zeolite materials may lead to a poor degradation performance during the treatment of pollutants with high molecular weight due to the diffusion limitation to the active sites located at the micropores [26]. Therefore, the accessibility of active sites for large molecules should be significantly increased in zeolites.

Diverse strategies have been developed to reduce the diffusion limitation by shortening the length of diffusion path, mainly including the introduction of mesopores and/or macropores into zeolite crystals (hierarchical zeolites), and decreasing crystal size [27]. Particularly, the emergence of zeolite nanosheets opens up unprecedented opportunities for promoting their adsorption, catalysis, and separation performances, due to the reduced diffusion path lengths, increased surface areas, and enhanced surface activity compared with their bulky counterparts [28,29]. Hensen et al. showed that Fe-MFI nanosheets (MFI zeolite framework, a code assigned by the International Zeolite Association corresponding to the ZSM-5 topology) were very active for the oxidation of benzene to phenol, compared with the conventional one, which were attributed to more isolated Fe centers and shorter diffusion pathways of Fe-MFI nanosheets than that of the conventional Fe-MFI [30]. However, despite significant advances in the synthesis of zeolite nanosheets and their applications in acid and redox catalysis [31], there is a conspicuous lack of research focusing on their potential in Fenton-like reactions, particularly regarding hierarchically structured zeolite nanosheets.

The present study developed hierarchical Fe-MFI nanosheets for efficient Fenton-like catalytic degradation of RhB. The synthesis consists of fluoride-mediated, seed-directed hydrothermal strategy to regulate nanosheet thickness and iron incorporation. Subsequent alkali treatment successfully etched abundant mesopores into the nanosheets, further enhancing the accessibility of active sites. Various characterization techniques were performed in detail to explore the correlation between Fenton-like degradation performance and catalysts structures, including morphology (the thickness of b-axis), pore structures, Fe structures, and Fe-dispersion of prepared catalysts.

## 2. Results and Discussion

### 2.1. Synthesis of the Fe-MFI Nanosheets with Different b-Axis Thicknesses

**Figure 1:**
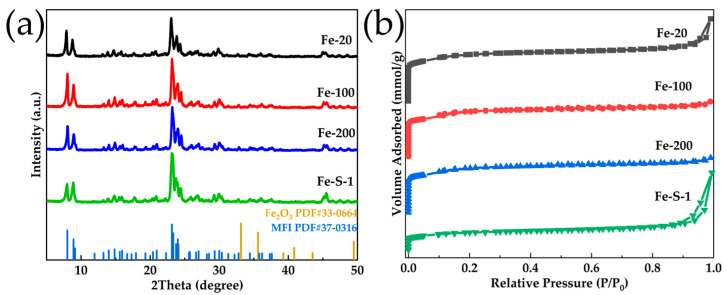
XRD patterns (**a**) and N_2_ adsorption–desorption curves (**b**) for Fe-S-1, Fe-20, Fe-100, and Fe-200 catalysts.

XRD patterns of Fe-x (Fe-20, Fe-100 and Fe-200) samples and comparison samples (Fe-S-1) are shown in Figure 1a. It is demonstrated that all samples display strong characteristic diffraction peaks belonging to the MFI topology. No XRD peaks belonging to bulk α-Fe_2_O_3_ phase (major reflections at 2*θ* = 33.2° and 35.7°) or any other iron oxide phases can be observed, suggesting that most of the Fe species were incorporated into the MFI framework or highly dispersed within zeolite channels [32]. This result indicated that Fe-MFI zeolites were successfully synthesized by a seed-induced hydrothermal method in the presence of NH_4_F.

N_2_ adsorption–desorption isotherms of the samples are displayed in Figure 1b. Distinct type-I isotherms can be observed for all samples, indicating the typical microporous materials. The textural parameters are summarized in Table 1. Fe-S-1 presented the lowest surface area and micropore volume, which may be due to the slow nucleation and growth rate in the absence of seed during the fluoride-mediate synthesis process. Fe–MFI nanosheets displayed similar specific surface area and micropore volume, while the mesopore volume of Fe-20 was larger than Fe-100 and Fe-200 that can be attributed to the stacking of nanosheets.

**Figure 2:**
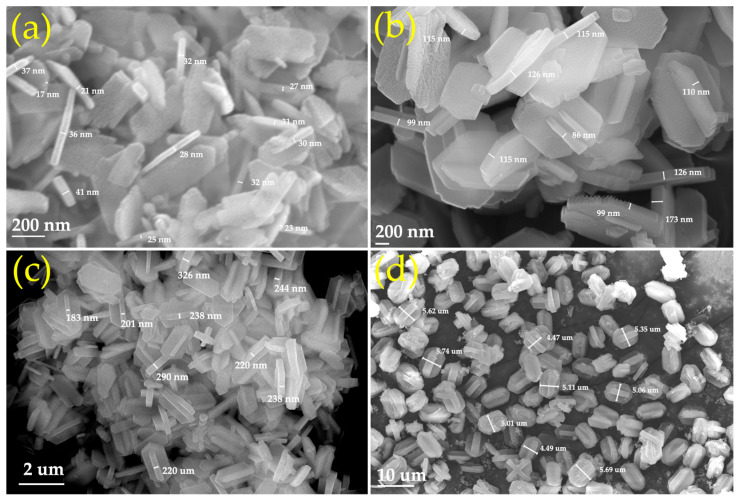
SEM images of Fe-20 (**a**), Fe-100 (**b**), Fe-200 (**c**), and Fe-S-1 (**d**).

The morphology of the samples was characterized by SEM. As shown in Figure 2, Fe-20, Fe-100, and Fe-200 illustrated well-defined sheet-like morphology with average thicknesses along the b-axis of ~29 nm, ~129 nm, and ~240 nm, respectively. The Fe–MFI nanosheets were obtained by a seed-induced hydrothermal method in the presence of NH_4_F. The addition of silicalite-1 seed overcomes the slow nucleation rate in the near-neutral solution; meanwhile, F^−^ is adsorbed on the [010] plane of crystals that inhibited the crystal growth along the b-axis [33]. The crystal size and adding amounts of seed determined the b-axis thickness, similar to the preparation of Fe–MFI nanosheets by a previous report. While Fe-S-1 presented a typical coffin-shaped morphology with a larger particle size of ~5.1 μm, which may be due to the slow nucleation rate in the presence of NH_4_F. It is well-known that nanosheet samples exhibited better diffusion performance compared with the bulk samples [34].

**Figure 3:**
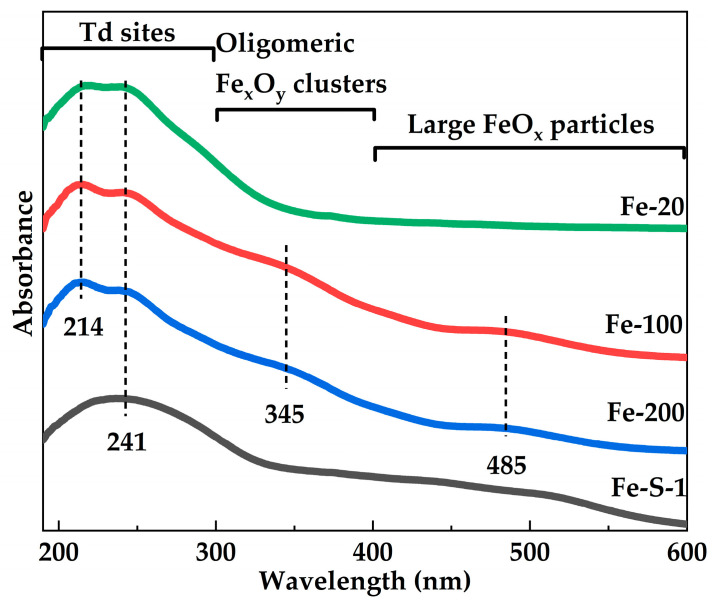
UV-Vis spectra of different samples.

UV-Vis diffuse reflectance spectroscopy was used to differentiate the various coordinate states of Fe species in those catalysts. As depicted in Figure 3, the Fe–MFI nanosheets exhibited two main absorbance peaks near 214 nm and 241 nm, which can be associated with the ligand to metal charge-transfer character derived from isolated 4-coordinated Fe^3+^ in the framework. The small band at 345 nm belongs to octahedral Fe^3+^ species in small oligomeric Fe*_x_*O*_y_* clusters located at the micropores or the surface. The bulk Fe-S-1 sample also displayed a broad band in the region of 400–600 nm, demonstrating the presence of FeO*_x_* nanoparticles [35]. It follows that adding seed suspension played the key effect in the incorporation of four-coordinated framework Fe^3+^ species. Fe–MFI nanosheets with different b-axis thicknesses presented similar Si/Fe ratios (Table 1); however, the intensities of the bands at 214 and 241 nm in the spectra of Fe-200 and Fe-100 were higher than that in the spectrum of Fe-20, suggesting many more framework 4-coordinated Fe^3+^ species existed in the two samples.

### 2.2. Fenton-like Oxidation Degradation of Rhodamine B by Fe-x Catalysts

**Figure 4:**
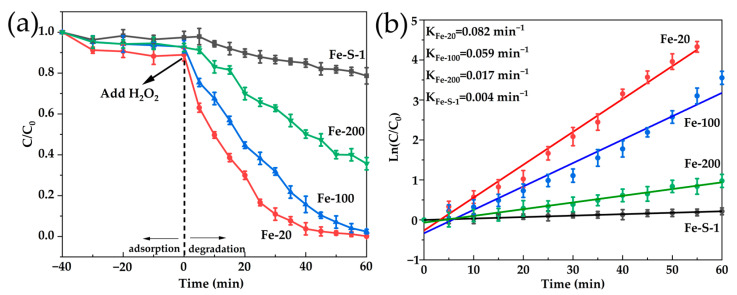
RhB degradation performances over Fe-S-1, Fe-200, Fe-100, and Fe-20: (**a**) RhB concentration as a function treatment time; (**b**) the corresponding kinetic plots. Experimental conditions: 40 mg/L RhB, 0.5 g/L catalyst, C_H2O2_ = 17.65 mmol/L.

The Fenton-like degradation performances of RhB over different Fe–MFI samples were evaluated. Prior to oxidation degradation, a magnetic stirring for 40 min in the absence of H_2_O_2_ was carried out to achieve the adsorption equilibrium of RhB on the catalyst powders. Figure 4a illustrates the degradation performances over different catalysts, the saturated adsorption amount of RhB decreased as Fe-20 > Fe-100 ≈ Fe-200 > Fe-S-1, corresponding to the variation in BET surface area of those samples (Table 1). For the subsequent oxidation degradation, the pseudo-first-order reaction can be observed (Figure 4b) and the concentration of RhB dropped exponentially with degradation time on those samples. The measured degradation rate over Fe-S-1 (k_Fe-S-1_) was 0.004 min^−1^. Surprisingly, Fe-MFI nanosheets exhibited much higher k values than Fe-S-1, demonstrating the better RhB degradation performances. The k values decreased in the order of Fe-20 (0.082 min^−1^) > Fe-100 (0.059 min^−1^) > Fe-200 (0.017 min^−1^), indicating that Fe-MFI nanosheets with a thicker b-axis facilitated the degradation of RhB. It is well-known that the doped transition metal ions in the framework structure of MFI can promote the dissociation of H_2_O_2_ to produce ·OH [36]. Considering the similar Si/Fe ratios (ICP results in Table 1) and Fe coordination (UV-Vis results in Figure 3), the increase in k values from 0.017 min^−1^ to 0.082 min^−1^ can be attributed to the decrease in the b-axis thickness from ~240 nm to ~29 nm that resulted into the significant improvement in RhB accessibility to Fe^3+/2+^ active sites.

### 2.3. Synthesis of the Hierarchical Fe-MFI Nanosheets

In order to further verify the influence of diffusion performance on RhB degradation performance, several hierarchical Fe-MFI nanosheets were prepared by a controlled alkali-treatment strategy using Fe-20 as the parent sample. The same NaOH concentration (0.2 M) but with different treatment times of 3, 10, and 30 min, was used to prepare Fe-20-*x*, where *x* represents the alkali-treatment time. The XRD patterns of the parent and the treated samples are shown in Figure S1, the relative crystallinity gradually decreases with prolonged treatment time that can be attributed to the creation of mesopores. The SEM images of Fe-20-x in Figure S2 illustrated that the sheet-like morphology changed a little after alkali treatment for 3 min and 10 min. Although the sheet-like structure was partially damaged by extending the treatment time to 30 min, a high solid yield of 57% was obtained. Previous studies found that the framework defects greatly decreased by introducing F^-^ during the synthesis process, which can inhibit the severe etching of nanosheets during alkali treatment [37].

**Figure 5:**
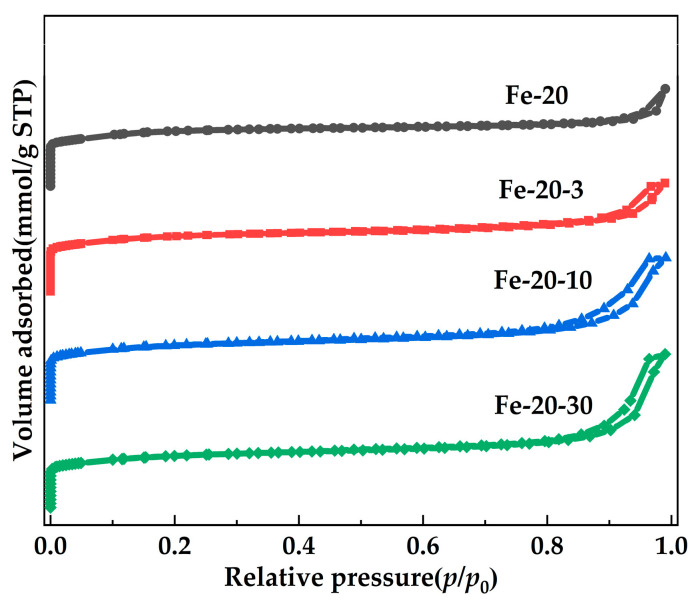
N_2_ adsorption–desorption curves for Fe-20, Fe-20-3, Fe-20-10, and Fe-20-30.

N_2_ physisorption results of the parent and the treated samples are shown in Figure 5. A similar nitrogen adsorption at low relative P/P_0_ indicated that the microporous structure was kept after alkali treatment. An obvious H2-type hysteresis loop at relative high pressures can be observed from the curves of the treated samples, suggesting the existence of mesopores [38]. As the alkali-treatment time increased, the hysteresis loop became more pronounced, indicating the creation of more mesopores (V_meso_ in Table 2).

**Figure 6:**
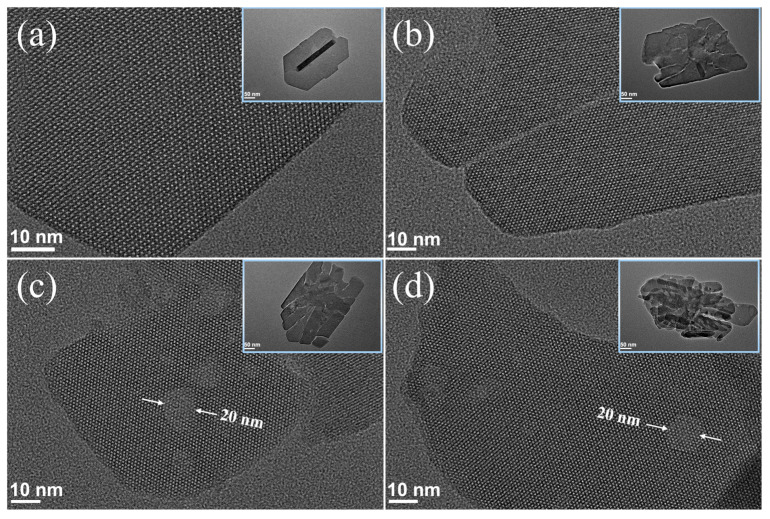
TEM images of Fe-20 (**a**), Fe-20-3 (**b**), Fe-20-10 (**c**), and Fe-20-30 (**d**).

The morphology and pore structure of the parent and the treated samples were further characterized by TEM. As shown in Figure 6, the TEM image of parent Fe-20 in Figure 6a exhibited a sheet-like morphology with well-defined lattice fringes, indicating its high crystallinity. After alkali treatment for 3 min, crack-like mesopores were formed and the sheet-like morphology remained largely intact (Figure 6b). The selective desilication of defective sites during alkali treatment and the special crystal growth mechanism of the nano-sheet structure in the presence of seed and NH_4_F can explain the generation of crack-like mesopores [33,39]. Further extending the alkali-treatment time to 10 min (Fe-20-10, Figure 6c) resulted in the formation of some isolated void-type mesopores having sizes around 20 nm, which was similar to the size of used silicalite-1 seed; thus, the formation of those mesopores may correlate with the dissolution of seed. Meanwhile, many more mesopores were generated by alkali treatment for 30 min and the nano-sheet structure was partially damaged, indicating excessive etching with a longer treatment time. The above results fully indicate that additional mesopores can be created by alkaline treatment of Fe-MFI nanosheets and the hierarchical Fe-MFI nanosheets were successfully obtained.

**Figure 7:**
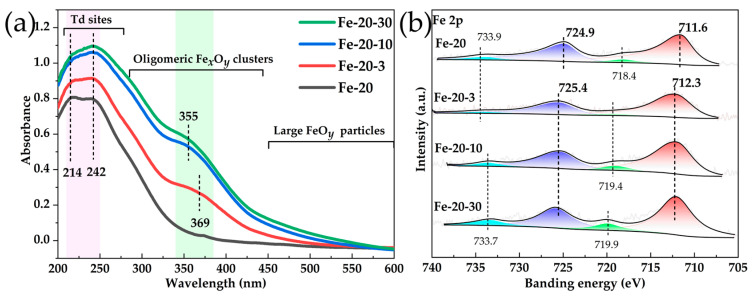
UV-Vis spectra (**a**) and Fe 2p XPS (**b**) for different samples.

To gain insight into the coordination state of Fe species during the alkaline treatment, UV-Vis spectra of the parent and the treated samples are depicted in Figure 7a. The parent (Fe-20) exhibits distinct absorption bands at 214 nm and 242 nm, which are attributed to the tetrahedral framework Fe^3+^ species. The intensities of these bands significantly increase after alkaline treatment. Moreover, the absorption peak intensities at 355 nm and 369 nm assigned to oligomeric Fe*_x_*O*_y_* clusters of the treated samples also significantly increased. Large FeO*_x_* nanoparticles (higher than 450 nm) can hardly be found in alkali-treated samples. The Si/Fe ratios (ICP data, Table 2) of alkali-treated Fe-20-x decreased with a longer treatment time, similar to the alkali treatment of Al-MFI zeolites.

The X-ray photoelectron spectra (XPS) were performed as a surface sensitive technique measuring chemical/electronic state of Fe species and the spectra of Fe 2p of the patent (Fe-20) and treated samples was shown in Figure 7b. All samples exhibit two strong peaks around 712 eV and 725 eV, corresponding to the Fe 2p_3/2_ and Fe 2p_1/2_ orbitals, respectively, along with two weak satellite peaks near 719 eV and 733 eV. The binding energy of these peaks was indicative of the presence of Fe^3+^ ions [40]. Notably, compared with pure iron oxide (BE was about 710.8 eV and 724.2 eV), Fe-20 patent showed a little positive shift in binding energy, which may be due to the higher electronegativity of silicon than Fe, further indicating the incorporation of Fe atoms into the zeolite framework [41]. More importantly, compared to Fe-20, the binding energy of the alkali-treated samples further increased, which may be due to the formation of oligomeric Fe*_x_*O*_y_* clusters [42], consistent with the UV-Vis results (Figure 7a).

The above results indicated that some framework Fe^3+^ species were transformed into oligomeric Fe*_x_*O*_y_* clusters during alkali treatment; however, the number of framework Fe^3+^ species kept almost unchanged due to the increase in Fe amount after alkali treatment.

### 2.4. Fenton-like Oxidation Degradation of Rhodamine B by Hierarchical of Fe-MFI Nanosheets

**Figure 8:**
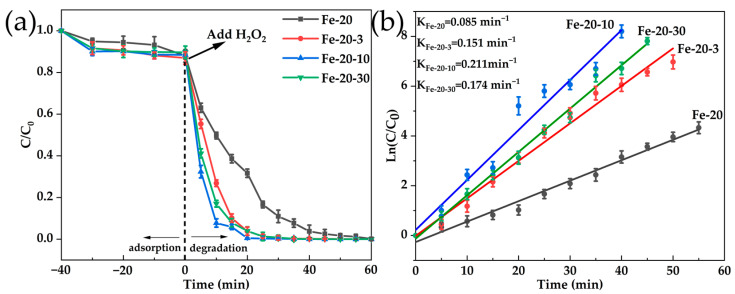
RhB degradation performances over the different catalysts (**a**) and fitting kinetic plots (**b**).

Similarly, the Fenton-like degradation of RhB was used to evaluate the catalytic performances of hierarchical Fe-MFI nanosheets. As shown in Figure 8, the alkali-treated nanosheets presented a similar higher RhB saturation adsorption capacity compared with the pristine sample that can be attributed to the introduction of additional mesopores into the nanosheets. The size of these pores is much larger than the molecular size of RhB, thereby enhancing the adsorption capacity of the catalyst. In the stage of oxidative degradation of RhB, the treated samples were also superior to that of the parent. The quasi-first order reactions were observed in Figure 8b; the k values decreased as k_Fe-20-10_ (0.211 min^−1^) > k_Fe-20-30_ (0.174 min^−1^) > k_Fe-20-3_ (0.151 min^−1^) > k_Fe-20_ (0.085 min^−1^). Obviously, Fe-20-10 exhibited the best degradation performance, which was 2.5 times that of the parent sample and 58.4 times that of the bulk Fe-S-1 (0.004 min^−1^). These results demonstrate that the creation of mesopores in Fe-MFI nanosheets plays an overwhelming role in the increase in RhB degradation performance. Moreover, Table S1 lists with other state-of-the-art Fenton-like Fe-based catalysts reported in recent years. Considering various reaction conditions (including catalyst dosage, RhB concentration, reaction time), hierarchical Fe-MFI nanosheets in this study should be a Fenton-like catalyst material with excellent degradation efficiency for RhB.

**Figure 9:**
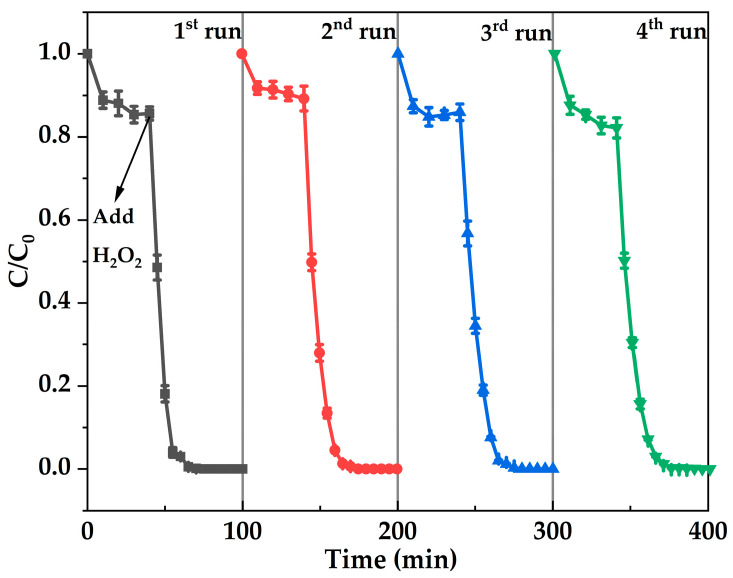
Recycling tests of Fe-20-10 for RhB degradation.

It is well-known that an important factor for catalysis is the reusability of catalysts. Therefore, the catalytic reusability of the Fe-20-10 catalyst was studied by recycling the catalytic degradation of RhB under the same conditions. The Fe-20-10 catalyst was recovered by washing with ethanol and deionized water at least three times, dried at 383K, and calcinated for the next run. It can be seen from Figure 9 that all the degradation percentages were higher than 98%, suggesting the better recyclability of Fe-20-10. A comparison of UV-Vis spectra between the fresh and regenerated Fe-20-10 catalysts is shown in Figure S3. It is found that the characteristic peak intensity corresponding to isolated tetrahedrally coordinated framework Fe species (214 nm and 242 nm) remains unchanged, whereas the peaks associated with non-framework oligomeric Fe*_x_*O*_y_* cluster (305 nm) decrease significantly. This indicates that the slight loss of activity after four catalytic cycles primarily stems from the leaching of non-framework Fe species. These results strongly demonstrate that the excellent reusability of the Fe-20-10 catalyst mainly originates from the stability of its active sites (isolated tetrahedrally coordinated framework Fe species).

### 2.5. Identifying of the Most Efficient Active Sites and Possible Reaction Process

It is well-reported that Fe species serve as one of the main active centers in Fenton-like reactions because of their ability to activate H_2_O_2_ to produce highly reactive oxidants (mainly hydroxyl radicals) which can degrade organic compounds in wastewater [43]. However, for the Fe-based zeolite catalysts, valency-controlled framework Fe^3+/2+^ species, oligomeric Fe*_x_*O*_y_* clusters, and FeO*_x_* nanoparticles [44,45] were proved to be the active species for Fenton-like reactions with H_2_O_2_. Therefore, in order to further identify the most efficient active sites in Fe-based zeolite catalysts, two reference samples of Fe/ZSM-5 and Fe/S-1 prepared by the impregnation method were also used for RhB degradation. XRD characterization confirms that both samples displayed the typical MFI topology (Figure S4). Fe/ZSM-5 exhibits a micron-sized bulk morphology, while Fe/S-1 sample displays a nanosheet morphology similar to that of the Fe-MFI sample (Figure S5). UV-Vis spectra in Figure S6 demonstrated that isolated Fe(III) ions with octahedral coordination (272 nm) dominated in the Fe/ZSM-5 sample, meanwhile some tetrahedral coordinated isolated framework Fe(III) species (210 nm) and external surface FeO*_x_* nanoparticles (540 nm) can also be observed. In contrast, more large FeO*_x_* nanoparticles can be found in Fe/S-1; meanwhile, the percentage of oligomeric Fe*_x_*O*_y_* clusters and framework Fe(III) ions in Fe/S-1 were much lower than that in Fe/ZSM-5. During the dynamic adsorption stage, the saturated adsorption amount of RhB over Fe/S-1 was much higher than that over Fe/ZSM-5, which can be ascribed to the nanosheet structure of the Fe/S-1. Interestingly, the fitting kinetic data indicated that isolated Fe(III) ions with octahedral coordination and tetrahedral coordinated isolated framework Fe(III) species were more efficient than other Fe species in the Fenton-like degradation reaction. It is also noted that the K value of the Fe-20 sample (0.082 min^−1^) was significantly higher than that of Fe/ZSM-5 (0.003 min^−1^), ruling out isolated Fe(III) ions with octahedral coordination as the primary active sites (Figure S7).

In order to further determine the most efficient active Fe sites in Fe-based zeolite for Fenton-like degradation reaction, hierarchical Fe-MFI nanosheets (Fe-20-10) were treated with HCl, aiming at removing non-framework Fe species. The obtained sample was denoted as Fe-20-10-acid. The band at 369 nm disappeared in the UV-Vis spectrum of Fe-20-10-acid (Figure S8), indicating selective removal of non-framework oligomeric Fe*_x_*O*_y_* clusters by mild HCl washing. Meanwhile, mild HCl treatment showed little influence on the framework Fe species. Fe-20-10 and Fe-20-10-acid presented a similar degradation performances (Figure S9) Therefore, it can be concluded that tetrahedral coordinated isolated framework Fe(III) species were more efficient in the Fenton-like degradation reactions.

**Figure 10:**
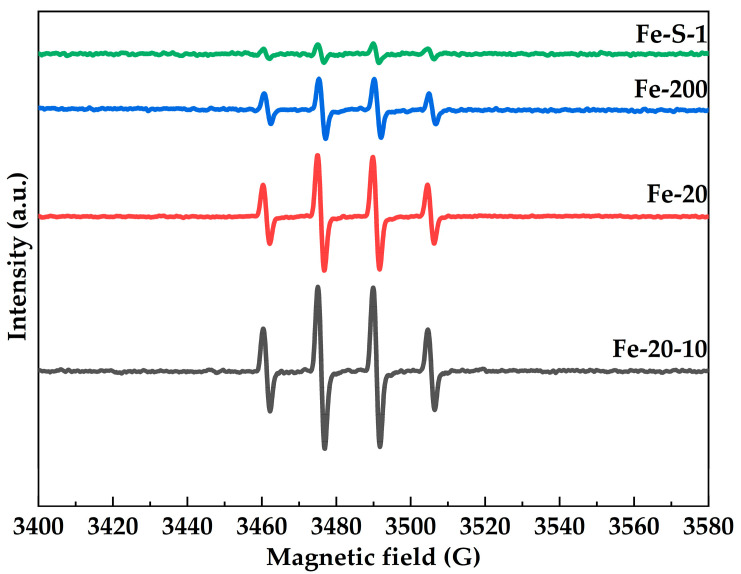
DMPO spin-trapping EPR spectra of Fe-S-1, Fe-200, Fe-20 and Fe-20-10.

It is well-known that the mechanism of Fenton-like degradation reaction of organic contaminant involved the activization of H_2_O_2_ by active center to generate reactive oxygen species (ROS) including OH· and HO_2_·. Subsequently, the organic contaminant was oxidized by ROS to H_2_O and CO_2_ [46]. The EPR spin-trap technique (5, 5-dimethyl-1-pyrroline N-oxide, DMPO, as the spin trapper) was employed to further confirm the ROS generated in Fe-MFI catalyst/H_2_O_2_ system. As shown in Figure 10, four-fold characteristic peaks of DMPO-·OH adducts with an intensity ratio of 1:2:2:1 were observed in all samples, which confirmed the formation of ·OH in these systems [47]. The intensity of such characteristic peaks increased as Fe-20-10 > Fe-20 > Fe-200 > Fe-S-1. This indicates that the unique nanosheet morphology possesses a larger specific surface area than the bulk sample (Fe-S-1), which exposes more Fe active sites, and a thinner thickness along the b-axis direction accelerates the diffusion efficiency of H_2_O_2_ within the channels and thus promoting ·OH generation. Moreover, hierarchical nanosheet sample (Fe-20-10) exhibited the highest ·OH yield, fully indicating the introduction of mesopores further enhanced the diffusion efficiency and the utilization of internal active Fe species, thereby increasing the ·OH production.

**Scheme 1:**
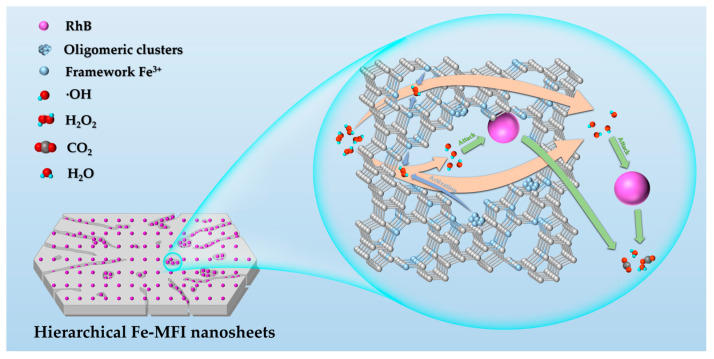
Schematic diagram of RhB degradation over hierarchical Fe-MFI nanosheets.

Based on the above research results, a possible pathway for Fenton-like degradation of RhB over the hierarchical Fe-MFI nanosheets was proposed as follows (Scheme 1): This process involved both adsorption and degradation stages. Since the molecular size of RhB (1.59 × 1.18 × 0.56 nm) is much larger than the micropore size of MFI zeolite (5.3 × 5.6 Å), the RhB mainly adsorbed on the external surface of Fe-MFI first. Fe-MFI nanosheets with larger external surface areas benefited from the adsorption of RhB. Meanwhile, the saturated adsorption capacity further increased by introducing additional mesopores. During the second step of Fenton-like degradation, the adsorbed organic contaminants were degraded or mineralized by the ROS generated from H_2_O_2_ and active Fe species. Hierarchical pore engineering in Fe-20-10 nanosheets enables efficient diffusion, driving enhanced accessibility of the active Fe sites located in the micropores and thus produced more ROS. As a result, a record-high degradation efficiency can be obtained.

## 3. Materials and Methods

### 3.1. Materials

Tetraethoxysilane (TEOS) and fluoride ammonium were obtained from Tianjin Yongda Chemical Regent Co., Ltd., Tianjin, China. Tetrapropylammonium bromide (TPABr) was sourced from Shanghai Jskchem Life Sciences Co., Ltd., Shanghai, China. Sodium hydroxide was supplied by Tianjin Bodhi Chemical Co., Ltd., Tianjin, China. Hydrogen peroxide (30 wt% in water) was provided by Tianjin Damao Chemical Regent Co., Ltd., Tianjin, China. Ammonium chloride was sourced from Beijing Chemical Works, Beijing, China. Rhodamine B was supplied by Tianjin Guangfu Fine Chemical Research Institute, Tianjin, China. Hydrochloric acid was obtained from Jinzhou Gucheng Chemical Regent Co., Ltd., Jinzhou, China. Tetrapropylammonium hydroxide (TPAOH, 25% in water) was provided by Sinopharm Chemical Reagent Co., Ltd., Shanghai, China. Nonahydrate iron(III) nitrate was obtained from Shanghai Aladdin Biochemical Technology Co., Ltd., Shanghai, China. Deionized water was produced in the laboratory. All the chemicals and reagents were used as received without further purification. Commercial ZSM-5 with a Si/Al molar ratio of 19 was purchased from the Nankai University Catalyst Factory, Tianjin, China.

### 3.2. Preparation of Seed Suspension

The molar composition of seed suspension was 1.0 SiO_2_: 0.15 TPAOH: 9 H_2_O. Firstly, 30 g of TEOS, 17.75 g of TPAOH, and 10.15 g of H_2_O were mixed and agitated at 303 K, and then the suspension was hydrothermally treated at 373 K for 48 h. The resulting suspension was used directly as the seed for subsequent synthesis of Fe-MFI nanosheet with b-axis thickness of 20 nm. The obtained seed suspension was denoted as Seed-1.

To prepare Seed-2, 20 g of TEOS and 20.74 g of TPAOH were mixed and stirred at 303 K, followed by hydrothermal treatment of the mixture at 353 K for 48 h. The resulting suspension was used directly as another seed for subsequent synthesis of Fe-MFI zeolite nanosheets with b-axis thickness of 100 nm and 200 nm. The molar composition of synthesis solution was 1.0 SiO_2_: 0.25 TPAOH: 9 H_2_O.

### 3.3. Preparation of Fe-MFI Nanosheets with Different Thicknesses

Fe-MFI zeolite nanosheets with a thickness of ~20 nm (referred to Fe-20) were synthesized using TEOS, TPABr, TPAOH, NH_4_F, Fe (NO_3_)_3_·9H_2_O, and Seed-1 as raw materials. In a typical synthesis, 1.20 g of TPABr, 37.80 g of deionized water, 4.82 g of TPAOH, 19.22 g of TEOS, and 1.96 g of Seed-1 were mixed at 313 K for 2 h. Next, a solution containing 2.68 g of NH_4_F and 32.94 g of deionized water was added dropwise to the aforementioned mixture. The new mixture was then stirred at 308 K for 0.5 h. Afterward, a solution containing 0.75 g of Fe (NO_3_)_3_·9H_2_O and 18.26 g of deionized water was added in above mixture and stirred at 308 K for 1.5 h. Finally, the resulting gel was placed into a Teflon-lined autoclave and heated at 443 K for 48 h with a rotating rate of 30 rpm. The molar ratio of the gel composition was 1.0 SiO_2_:0.0125 Fe_2_O_3_:0.237 TPAOH:0.045 TPABr:0.8 NH_4_F:50 H_2_O. After crystallization, the products were filtered, washed, and then dried overnight at 373 K. Finally, the obtained powder was calcined in air at 803 K for 6 h.

Similarly, Fe-MFI nanosheets with thickness of 100 nm (Fe-100) and 200 nm (Fe-200) were synthesized using 3.69 g and 1.85 g of Seed-2, respectively. In contrast, a comparison sample of Fe-S-1 was synthesized by a similar route without adding any seeds. Pure S-1 nanosheet a with a thickness of ~20 nm (referred to S-1) was also synthesized by a similar route of Fe-20 sample without adding Fe source (Fe (NO_3_)_3_·9H_2_O).

### 3.4. Preparation of Hierarchical Fe-MFI Nanosheets

The Fe-20 was alkali-treated using the following procedure: First, 2 g of Fe-20 was added to 60 mL of 0.2 M NaOH aqueous solution and stirred at 353 K for 3, 10, or 30 min. After alkali treatment, the slurry was rapidly cooled to room temperature in an ice bath, filtered, and washed with deionized water until a neutral pH was reached. The solid was dried at 373 K overnight and then calcined in static air at 803 K for 3 h to obtain the Na-form hierarchical Fe-MFI nanosheets. The samples were labeled as Na-Fe-20-*x* (where *x* represents the alkali-treatment time).

The Na-form hierarchical Fe-MFI nanosheets were ion-exchanged three times with 1 M NH_4_Cl solution (liquid-to-solid ratio: 20 mL/g) for 1.5 h each under stirring. After filtration and washing with deionized water, the material was dried and calcined in static air at 803 K for 3 h to obtain the H-form hierarchical Fe-MFI nanosheets. The final samples were designated as Fe-20-3, Fe-20-10, and Fe-20-30 based on the alkali-treatment time.

The reference samples Fe/ZSM-5 and Fe/S-1 with Si/Fe molar ratios of 40 were synthesized by wet impregnation method. Typically, for the synthesis of Fe/ZSM-5, 0.16 g Fe(NO_3_)_3_·9H_2_O was dissolved in 0.7 g deionized water. Then, 1 g of commercial ZSM-5 zeolite with Si/Al ratio of 19 was added to the prepared solution and stirred for 24 h at room temperature and heated under stirring to evaporate the water till dryness. The resulting solids were then calcined at 823 K for 5 h. Fe/S-1 was prepared using a similar procedure as described. Typically, 0.168 g Fe(NO_3_)_3_·9H_2_O was dissolved in 1.0 g deionized water. Then, 1 g of S-1 nanosheet sample was added to the prepared solution and stirred for 24 h at room temperature and heated under stirring to evaporate the water till dryness. The resulting solids then calcined at 823 K for 5 h. For the synthesis of Fe-20-10-acid, 0.6 g of Fe-20-10 sample was added in 0.2 mol/L HCl solution (12 mL) and stirred thoroughly at 348 K for 2 h. The solid products were obtained by repeatedly filtering and washing with deionized water until neutral, drying at 373 K overnight, and calcination at 823 K for 5 h.

### 3.5. Characterizations Instruments

The XRD characterization of the synthesized catalyst was performed using a D/MAX-2500 X-ray diffractometer (manufactured by Rigaku Corporation, Kyoto, Japan). The analysis was conducted with Cu Kα radiation under operational conditions of 40 kV tube voltage and 100 mA tube current. The scanning range was set at 5–50° with a scanning rate of 8°/min.

The morphology of the material was characterized using a scanning electron microscope (SEM, Carl Zeiss, Jena, Germany) operating at an acceleration voltage of 200 kV.

The morphology and structure of the catalyst were observed using transmission electron microscopy (TEM, FEI Tecnai G2 F20, Hillsboro, OR, USA). For sample preparation, the powder was ultrasonically dispersed in anhydrous ethanol, and the resulting suspension was drop-cast onto a copper grid (carbon film support) followed by vacuum drying.

Nitrogen adsorption–desorption isotherms were measured at 77 K using a Micromeritics ASAP-2020 analyzer (Norcross, GA, USA). Prior to analysis, each sample was degassed under vacuum (10^−3^ Pa) at 623 K for 10 h. The specific surface area (SBET) was calculated from the adsorption data in the relative pressure (P/P_0_) range of 0.01–0.04 using the Brunauer–Emmett–Teller (BET) equation.

Inductively coupled plasma optical emission spectroscopy (ICP-OES, Perkin Elmer Optima2000DV, Waltham, MA, USA) was used for the elemental analysis of silicon and Fe content for all reported catalysts.

The coordination state of iron was analyzed using a UV-550 UV-visible spectrophotometer (Jasco Company, Tokyo, Japan), with barium sulfate (BaSO_4_) powder serving as the reference sample.

The reactive ·OH radicals were determined by 5, 5-dimethyl-1-pyrroline N-oxide (DMPO) spin-trapping EPR technology (Chinainstru & Quantumtech Co., Ltd., Beijing, China). Before the test, 10 mg of catalyst was first dispersed in 1 mL of deionized water and 100 μL of 3% hydrogen peroxide. After stirring for 2 min and subsiding for 1 min, 20 μL of this mixed solution was taken and injected to 500 μL DMPO (100 mM) immediately. The obtained solution was transferred to a 100 µL capillary tube, which was then fixed in the resonant cavity of the spectrometer. DMPO trapping measurements were detected at room temperature and recorded by one scan.

X-ray photoelectron spectroscopy (XPS) was conducted on a Thermo Scientific TM ESCALAB 250Xi (produced by Thermo Fisher Scientific, Waltham, MA, USA) spectrometer equipped with a monochromatic Al Kα X-ray source (1486.6 eV) operating at 150 W. Samples were analyzed under vacuum (P < 10−8 mbar) with a pass energy of 100 eV (survey scans) or 50 eV (high-resolution scans). All peaks would be calibrated with C1s peak binding energy at 284.8 eV for adventitious carbon.

### 3.6. Heterogenous Fenton-like Degradation

The Fenton-like degradation of RhB was carried out. Overall, 0.05 g of the prepared sample and 100 mL of RhB solution were added to a 200 mL beaker. The beaker was placed in a thermostatic water bath with continuous stirring for 100 min. During the initial 40 min of reaction, aliquots were collected every 10 min. Those samples were filtered through 0.22 μm membranes and stored in 10 mL test tubes. Subsequently, 0.2 mL of H_2_O_2_ was added dropwise, and the reaction continued for an additional 60 min with sampling every 5 min. After completion, the absorbance of solutions was measured by UV-Vis spectrophotometry (TU-1810, Beijng Puxi General Instrument Co., Ltd., Beijing, China) at the wavelength of 554 nm. The degradation behavior of RhB over the catalyst during the last 60 min was analyzed using the pseudo-first-order kinetics model formulated by Equations (1) and (2),

ln(C_0_ − C) = lnC_0_ − Kt (1)

(2) $$\ln\frac{C_{0}}{C}\;=\;\mathrm{Kt}$$

where C_0_ presents the absorbance of unreacted RhB solution, C denotes the absorbance during the reaction, and K is the kinetic rate constant.

## 4. Conclusions

In this study, the microporous Fe-MFI nanosheets with b-axis thickness of ~29, ~129, and ~240 nm was first synthesized via a seed-directed hydrothermal method in the presence of NH_4_F for the degradation of Rhodamine B by Fenton-like reaction. Their degradation performance improved by decreasing the b-axis thickness. Fe-20 with the b-axis thickness of ~29 nm exhibited a higher degradation rate of 0.082 min^−1^ which was 58.4 times higher than that of bulk Fe-S-1, which was attributed to shorter diffusion pathways and a larger specific surface area, both enhancing the catalytic degradation efficiency. More importantly, to further improve the diffusion property, the hierarchical Fe-MFI nanosheets were synthesized by a subsequent NaOH treatment. It is also found that abundant mesopores were created into Fe-MFI nanosheet and the number of framework Fe species kept almost unchanged. The degradation rate over hierarchical Fe-MFI nanosheets further increased to 0.211 min^−1^. The optimal catalyst also displayed similar high degradation performances in four recycling tests. By comparing the degradation performances of Fe-MFI nanosheets, supported Fe/ZSM-5, Fe/S-1, and acid-treated Fe-MFI nanosheet, it was confirmed that highly dispersed framework tetrahedral Fe(III) species served as the efficient active sites for RhB degradation. This strategy provides an efficient method to the synthesis of hierarchical zeolite nanosheet, which may act as a promising candidate for practical advanced oxidation processes in wastewater purification systems.

## Figures and Tables

**Table 1:** Texture properties of Fe-20, Fe-100, Fe-200, and Fe-S-1.

Sample	Si/Fe Ratio ^a^	Surface Area (m^2^·g^−1^)		Pore Volume (cm^3^·g^−1^)		
		Total ^b^	Exter ^c^	Total ^c^	Micro ^c^	Meso ^d^
Fe-20	36	425	79	0.31	0.14	0.17
Fe-100	36	396	89	0.21	0.13	0.08
Fe-200	37	396	87	0.21	0.13	0.08
Fe-S-1	32	171	56	0.28	0.05	0.23

^a^ Determined by ICP-OES; ^b^ calculated by the BET method; ^c^ calculated by t-plot method; ^d^ calculated by subtracting micropore volume from total pore volume.

**Table 2:** Texture properties of Fe-20, Fe-20-3, Fe-20-10, and Fe-20-30.

Sample	Si/Fe Ratio ^a^	Surface Area (m^2^·g^−1^)		Pore Volume (cm^3^·g^−1^)		
		Total ^b^	Exter ^c^	Total ^c^	Micro ^c^	Meso ^d^
Fe-20	36	425	79	0.31	0.14	0.17
Fe-20-3	31	426	96	0.34	0.13	0.21
Fe-20-10	26	426	110	0.45	0.13	0.32
Fe-20-30	25	404	104	0.48	0.12	0.36

^a^ Determined by ICP-OES; ^b^ calculated by the BET method; ^c^ calculated by t-plot method; ^d^ calculated by subtracting micropore volume from total pore volume.

## Data Availability

The original contributions presented in the study are included in the article (and Supplementary Materials), further inquiries can be directed to the corresponding authors.

## Supplementary Materials

The following supporting information can be downloaded at: https://www.mdpi.com/article/10.3390/molecules30194030/s1, Figure S1: XRD patterns of Fe-20, Fe-20-3, Fe-20-10 and Fe-20-30.; Figure S2: SEM images of Fe-20-3 (**a**), Fe-20-10 (**b**) and Fe-20-30 (**c**); Figure S3: UV-Vis spectra of the fresh and the used Fe-20-10; Figure S4: XRD patterns for Fe/S-1 and Fe/ZSM-5; Figure S5: SEM images of Fe/ZSM-5 (**a**) and Fe/S-1 (**b**); Figure S6: UV-Vis spectra of Fe/S-1 and Fe/ZSM-5; Figure S7: Degradation over the different catalysts for Fe/S-1 and Fe/ZSM-5 (**a**) and their kinetic plots (**b**); Figure S8: UV-Vis spectra of Fe-20-10 and Fe-20-10-acid; Figure S9: UV-Vis spectra (**a**) and kinetic plots **b**) of Fe-20-10 and Fe-20-10-acid; Table S1: Comparison of the Fenton-like performance between some Fe-based catalysts and Hierarchical Fe-MFI nanosheets, see [48,49,50,51,52].
