# Supplementary material for: Construction of Hierarchical Fe-MFI Nanosheets with Enhanced Fenton-like Degradation Performance
Source: Molecules. 2025 Oct 9;30(19):4030. doi: 10.3390/molecules30194030 (PMC12525659; doi:10.3390/molecules30194030)
Supplement: Supplementary file 1 [file molecules-30-04030-s001.zip › molecules-3824245-supplementary.pdf]

Supporting Information

Construction of Hierarchical Fe-MFI Nanosheets with Enhanced Fenton-like Degradation Performance

Haibo Jiang 1, Lin Xu 1, Qingrun Meng 1,*, Xu Feng 1, Junxuan Wang 1, Yankai Li 1 and Junjie Li 2,*

[1] Key Laboratory of Energy Chemical and Nano-Catalysis of Liaoning Province, School of Chemical and Envi-ronmental Engineering, Liaoning University of Technology, Jinzhou 121001, China

[2] Dalian Institute of Chemical Physics, Chinese Academy of Sciences (CAS), Dalian 116023, China

* Correspondence: meng081015@lnut.edu.cn (Q.M.); lijj@dicp.ac.cn (J.L.)

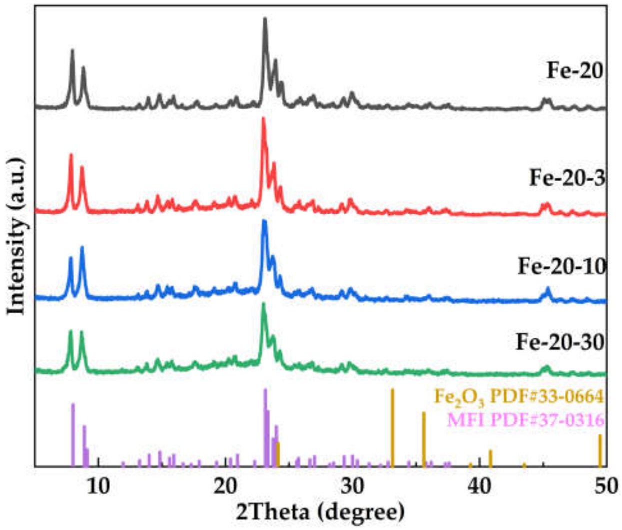

Figure S1. XRD patterns of Fe-20, Fe-20-3, Fe-20-10 and Fe-20-30.

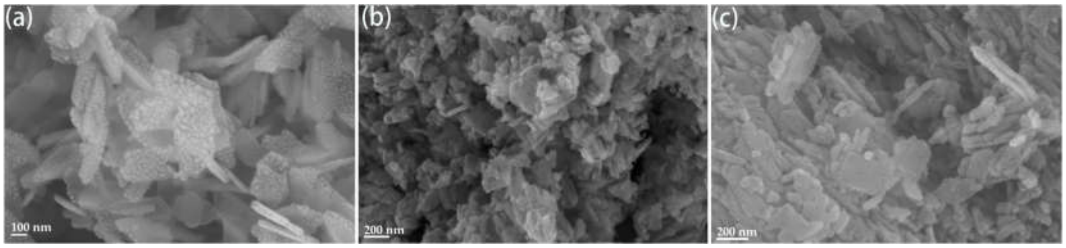

Figure S2. SEM images of Fe-20-3 (**a**), Fe-20-10 (**b**) and Fe-20-30 (**c**).

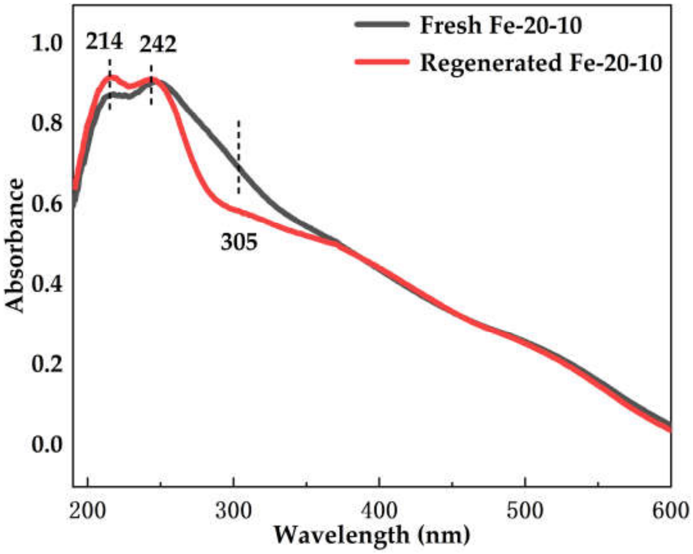

Figure S3. UV-Vis spectra of the fresh and the regenerated Fe-20-10.

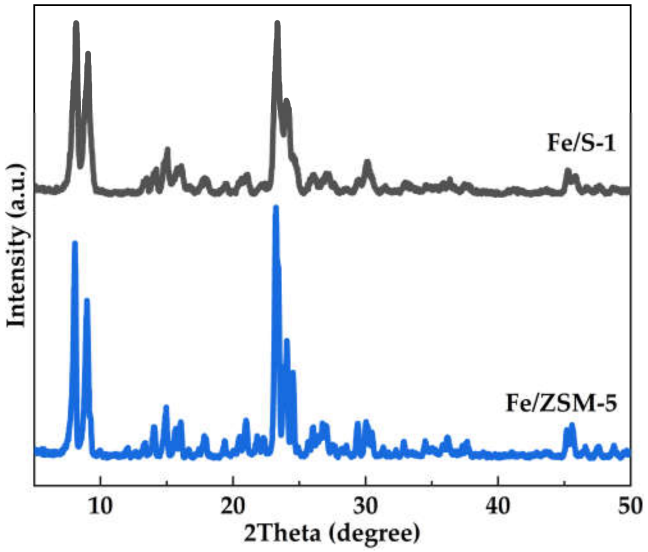

Figure S4. XRD patterns for Fe/S-1 and Fe/ZSM-5.

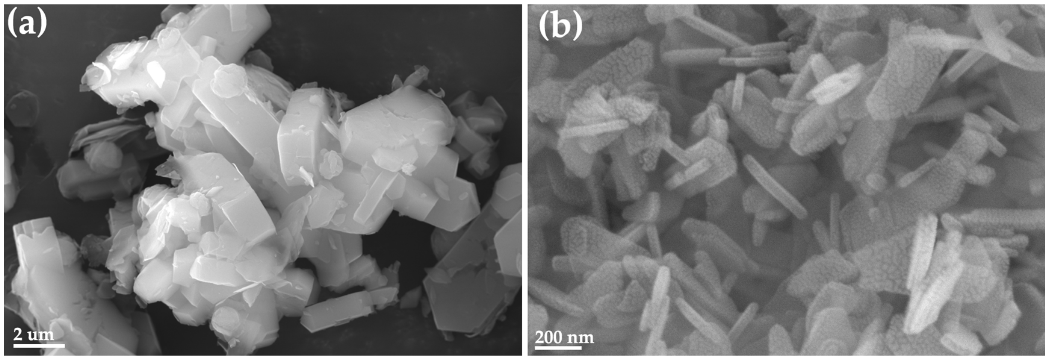

Figure S5. SEM images of Fe/ZSM-5 (**a**) and Fe/S-1 (**b**).

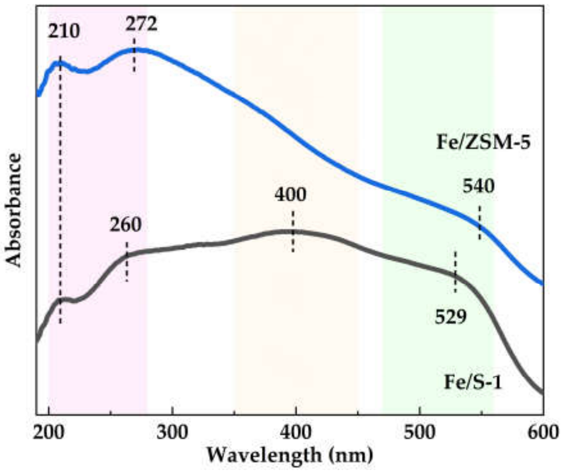

Figure S6. UV-Vis spectra of Fe/S-1 and Fe/ZSM-5.

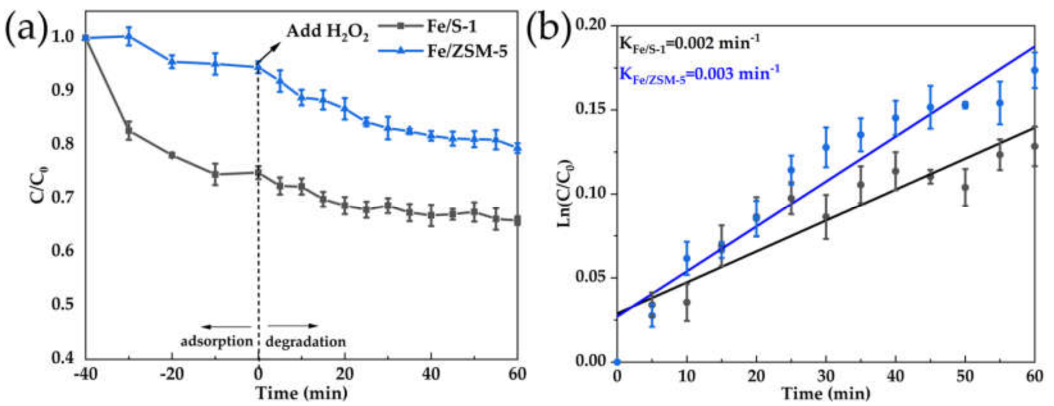

Figure S7. Degradation over the different catalysts for Fe/S-1 and Fe/ZSM-5 (**a**) and their kinetic plots (**b**).

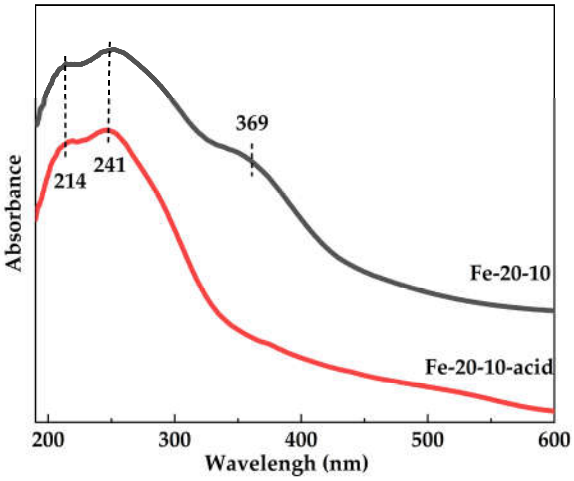

Figure S8. UV-Vis spectra of Fe-20-10 and Fe-20-10-acid.

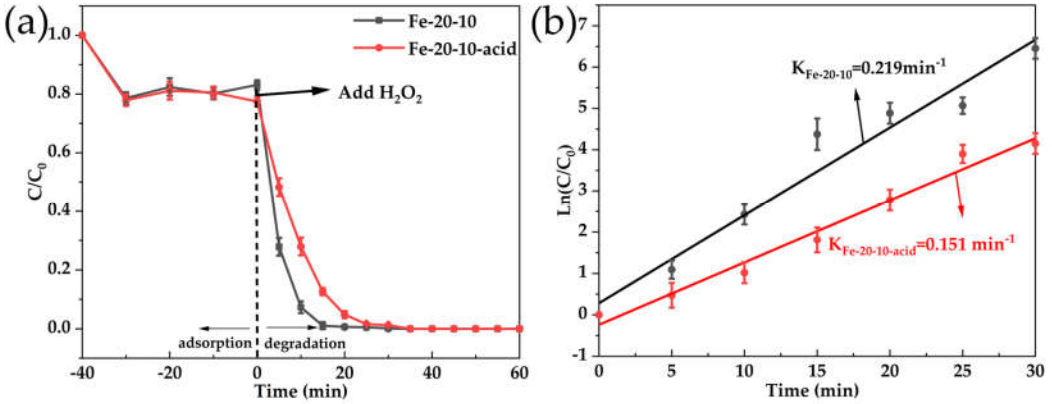

Figure S9. UV-Vis spectra (**a**) and kinetic plots (**b**) of Fe-20-10 and Fe-20-10-acid.

Table S1. Comparison of the Fenton-like performance between some Fe-based catalysts and Hierarchical Fe-MFI nanosheets.

Catalysts	Catalyst Dosage g/L	RhB Concentration mg/L	Reaction Time min	Degradation Efficiency %	Ref.
Hierarchical Fe-MFI nanosheets	0.5	40	20	100.0	This work
α/γ-Fe_2O_3	0.2	1	12	99.2	[1]
FeNPs	1	20	180	99.1	[2]
FeZSM-5	1	30	40	>97	[3]
Fe-supported perlite	0.6	25	110	99.6	[4]
Fe_2O_3 loaded inside halloysite nanotubes	0.5	30	120	92.9	[5]
Fe_3O_4@MHC	0.17	10	60	97	[6]

References

1. Magomedova, A.; Isaev, A.; Orudzhev, F.; Sobola, D.; Murtazali, R.; Rabadanova, A.; Shabanov, N.S.; Zhu, M.; Emirov, R.; Gadzhimagomedov, S.; et al. Magnetically Separable Mixed-Phase α/γ-Fe2O3 Catalyst for Photo-Fenton-like Oxidation of Rhodamine B. *Catalysts* **2023**, *13*, 872, doi:10.3390/catal13050872.
2. Corrêa, C.R.R.; De Siqueira, A.B.; Matos Lopes, P.R.; Ambrosio, J.A.R.; Simioni, A.R.; De Vasconcelos, L.G.; De Morais, E.B. Green Synthesis of Iron Nanoparticles from the Baru (*Dipteryx Alata*) Endocarp Extract for the Efficient Removal of Rhodamine B and Caffeine from Water through the Heterogeneous Fenton Process. *AQUA — Water Infrastructure, Ecosystems and Society* **2024**, *73*, 771–789, doi:10.2166/aqua.2024.300.
3. Deng, S.; Zhang, X.; Lv, G.; Zhai, Y.; Yang, Z.; Zhu, Y.; Li, H.; Wang, F. Influence of Zeolite Carriers on the Dyes Degradation for Framework Fe-Doped Zeolite Catalysts. *J Sol-Gel Sci Technol* **2019**, *91*, 54–62, doi:10.1007/s10971-019-05030-2.
4. Oliveira Aguiar Perez, J.; Tuono Martins Xavier, G.; Felix Bitencourt, G.; Dos Santos Andrade, L.; Alves Carvalho, W.; Jesus Olortiga Asencios, Y. Fe-Supported Perlite Applied for the Degradation of Rhodamine B Dye by Heterogeneous Fenton Reaction. *JSSE* **2025**, *2*, 14, doi:10.34024/jsse.2024.v2.19444.
5. Li, Y.; Zhou, J.-Q.; Xu, H.-Y.; Dong, L.-M.; Cao, M.-C.; Shan, L.-W.; Jin, L.-G.; He, X.-L.; Qi, S.-Y. Comparative Studies on Fenton-like Reactions Catalyzed by Fe3O4 Loaded inside and Outside Halloysite Nanotubes for the Removal of Organic Pollutants. *Front. Mater. Sci.* **2024**, *18*, 240673, doi:10.1007/s11706-024-0673-0.
6. Zhang, C.; Zeng, J.; Ouyang, Z.; Li, X.; Lin, L.; Peng, Y.; Gong, X. Reuse of Microalgae Residue after Oil Production as a Fenton-like Catalyst in Wastewater Treatment: Catalytic Performance and Mechanism. *Journal of Water Process Engineering* **2023**, *55*, 104092, doi:10.1016/j.jwpe.2023.104092.
